# The JAK-STAT Signaling Pathway in Epilepsy

**DOI:** 10.2174/1570159X21666221214170234

**Published:** 2023-08-15

**Authors:** Huaiyu Sun, Di Ma, Yu Cheng, Jiaai Li, Wuqiong Zhang, Ting Jiang, Zhaoran Li, Xuewei Li, Hongmei Meng

**Affiliations:** 1 Department of Neurology and Neuroscience Center, The First Hospital of Jilin University, Changchun, China;; 2 Department of Radiology, The First Hospital of Jilin University, Changchun, China

**Keywords:** Cytokines, epilepsy, GABA, JAK-STAT, seizures, signaling pathways

## Abstract

Epilepsy is defined as spontaneous recurrent seizures in the brain. There is increasing evidence that inflammatory mediators and immune cells are involved in epileptic seizures. As more research is done on inflammatory factors and immune cells in epilepsy, new targets for the treatment of epilepsy will be revealed. The Janus kinase-signal transducer and transcriptional activator (JAK-STAT) signaling pathway is strongly associated with many immune and inflammatory diseases, At present, more and more studies have found that the JAK-STAT pathway is involved in the development and development of epilepsy, indicating the JAK-STAT pathway’s potential promise as a target in epilepsy treatment. In this review, we discuss the composition, activation, and regulation of the JAK-STAT pathway and the relationship between the JAK-STAT pathway and epilepsy. In addition, we summarize the common clinical inhibitors of JAK and STAT that we would expect to be used in epilepsy treatment in the future.

## 1 INTRODUCTION

Epilepsy is one of the most common neurological disorders in the world. People with epilepsy also develop learning disabilities, memory problems, autism, and other neuropsychiatric disorders that seriously affect their quality of life [1]. The causes of epilepsy include genetics, infections, metabolism, immunity, and structure. However, for most patients, the cause of their epilepsy remains unknown [2]. Non-surgical treatment (surgery or vagus nerve stimulation) is currently available for a small number of patients, but antiepileptic drugs remain the mainstay of treatment for epilepsy in most patients. However, approximately 1/3 of people with epilepsy develop resistance to antiepileptic drugs [3]. In addition, side effects of some antiepileptic drugs are unavoidable during treatment. As a result, new treatments are urgently needed, that can suppress seizures and avoid unnecessary side effects.

A major function of the JAK-STAT pathway is to regulate growth, hormone release, inflammation, and tumor growth. The dysregulation of this pathway can lead to diseases such as cancer, inflammation, and neurodegenerative diseases. The regulation of JAKs and STATs by their phosphorylation or negative regulation is the main mechanism affecting JAK-STAT activity. The results of several experiments and clinical trials suggest that small-molecule JAK inhibitors (Jakinibs), as well as STAT inhibitors targeting JAK-STAT signaling, can reduce inflammatory responses caused by certain diseases, such as autoimmune and inflammatory diseases [4, 5].

Inflammatory and immune factors contribute greatly to the development of epilepsy through neuroinflammation. The release of inflammatory factors and destruction of the blood-brain barrier caused by inflammation, and the activation of astrocytes and microglia, can cause epilepsy, which further causes inflammation and promotes the development of epilepsy. The JAK-STAT signaling pathway primarily mediates inflammation and immune responses. Currently, trials of JAK-STAT pathway inhibitors have shown good therapeutic efficacy and they have been partially approved for clinical use. In recent years, an increasing number of studies have shown that the JAK-STAT pathway is involved in the development of epilepsy, and inhibition of JAK-STAT activity may lead to epilepsy treatment and further generation of new small anti-inflammatory molecules [6-8].

## 2 EPIDEMIOLOGY OF EPILEPSY

Epilepsy is a chronic neurological disorder that affects millions of people around the world. The number of epilepsy patients worldwide exceeds 70 million, three-quarters of whom live in resource-poor countries with a lack of medical services and treatment [9, 10]. The prevalence of epilepsy varies by country and region. The median prevalence of active epilepsy was 4.9 per 1,000 for developed countries and 12.7 per 1,000 and 5.9 per 1000 in rural and urban studies in developing countries [11]. Prevalence studies from 12 resource-poor countries reported a median prevalence of active epilepsy of 12.4 per 1000 [12]. The ILAE 2017 classification shows that there are six etiologies of epilepsy: structural, genetic, infectious, metabolic, immune, and unknown. In a study of 112,744 children aged 3 to 13, there were 606 children with epilepsy (CWE), among whom focal epilepsies (59% of CWE) and generalized epilepsies (35% of CWE) were the most common. Based on the etiological classification, epilepsy was classified as unknown in 229 per 100,000 children (40% of CWE). The genetic etiology included 183 cases per 100,000 children (32% of CWE) and structural etiology in 141 cases per 100,000 children (25% of CWE). Metabolic and infectious factors accounted for only 1% and 2% of the CWE, respectively. However, none of the 606 CWE had an immunological cause of epilepsy [13, 14]. In another study of 653 adults with epilepsy, there were 458 cases of focal epilepsy, 155 cases of generalized epilepsy, and 11 cases of combined focal and general epilepsy (1.7%). 29 (4.4%) of the patients had no known cause of epilepsy. Of the 653 patients with epilepsy, there were 169 cases with genetic or presumed genetic causes, 25.9%, and 179 cases with structural causes, 27.4% infectious and immunological factors accounted for 5.8% and 0.3% of people with epilepsy, respectively, and there were no metabolic causes of epilepsy in this study [13, 15]. The age-specific incidence curve for epilepsy presents a typical U-shaped (bimodal) pattern with high incidence in children and the elderly [16, 17]. At present, the sex distribution of epilepsy varies in different studies, and some studies have shown a higher proportion of men than women with epilepsy [18, 19], and in other studies, there is no difference in the incidence of epilepsy by sex [20, 21]. In addition, the incidence in children is lower in boys than in girls [22].

## 3 IMMUNOLOGY IN THE ETIOLOGY OF EPILEPSY

The International League Against Epilepsy (ILAE) defines six etiological categories: structural, genetic, infectious, metabolic, immune, and unknown. In some patients, there may be more than one cause of epilepsy.

Of the six causes, infection was the most common, responsible for seizures and persistent epilepsy. Infectious causes include bacterial infections (typically bacterial meningitis and tuberculosis), viral infections (herpes simplex), and parasitic diseases (toxoplasmosis and malaria) [23]. Several factors contribute to epilepsy after infection, including the source of infection, the severity of brain injury, age, and genetics. During the incubation period between infection and seizures, various brain changes can occur, including impaired integrity of the blood-brain barrier (BBB), neuronal overexcitation, neuronal loss, and gliosis, which may eventually lead to spontaneous recurrent seizures. The treatment of post-infection epilepsy is similar to that of other symptomatic epilepsy, and antiepileptic drugs are selected according to symptomatology. In the treatment of epilepsy, clinicians need to simultaneously search for infectious causes of seizures, understand local endemic diseases, epidemics, and rationally use antibacterial drugs. However, some antimicrobial agents may induce seizures and increase or decrease the plasma levels of antiepileptics. Therefore, these issues should be considered when using antimicrobial agents [24].

Infection and immune factors can cause inflammation in the body. More and more studies have shown that neuroinflammation leads to epilepsy development. As a consequence of neuroinflammation, peripheral immune cells can penetrate the BBB, and consequently release pro-inflammatory cytokines (PICs), including interleukin-1 β (IL-1β), IL-1α, IL-6, IL-17, IL-18, Tumor necrosis factor-α (TNF-α), and interferon-γ (IFN-γ) [25]. Simultaneously, inflammatory factors in peripheral blood can enter the brain through the damaged BBB [26]. Neurons, astrocytes, pericytes, perivascular microglia, and perivascular microglia play roles in the formation and maintenance of the BBB. In the central nervous system (CNS), the BBB protects against invasive pathogens and maintains stability [27, 28]. The inflammation of the CNS causes an increase in reactive oxygen species (ROS) and oxidative stress in the brain, which increases susceptibility to seizures and exacerbates the inflammatory state of the brain by PICs, such as transforming growth factor-β (TGF-β), and prostaglandin E2 (PGE2), which stimulate astrocytes and modulate glutamate release, resulting in increased excitability [29, 30]. Activated astrocytes and microglia are found in people and animals with epilepsy [31, 32]. Astrocytes in the brains of epileptic patients modulate cell excitability by regulating extracellular K^+^ concentration and glutamate reuptake, and microglia can influence neuronal excitability by increasing the sodium current density [33, 34]. Microglias are activated earlier than astrocytes, and cytokines released by microglia subsequently induce astrocyte activation, function, and proliferation [35, 36].

Epilepsy may also be associated with the immune system. Epilepsy is promoted by immune cells activated by natural immunity, adaptive immunity, and inflammatory mediators. The most common manifestations of antibody-associated autoimmune epilepsy are borderline encephalitis with seizures and chronic or acute seizures [37-39]. Among these, autoimmune encephalitis and epilepsy are associated with intracellular antibodies (glutamic acid decarboxylase 65, anti-neuronal nuclear antibodies-1, and Ma) and neuronal surface antibodies (voltage-gated potassium channel complex, N-methyl-d-aspartate receptor, alpha-amino-3-hydroxy-5-methyl-4-isoxazole-propionic acid, gamma-aminobutyric acid, and metabotropic glutamate receptor 5) [40]. Currently, the diagnosis of autoimmune epilepsy is based on clinical features, magnetic resonance imaging (MRI), and cerebrospinal fluid (CSF) analysis. The early identification of immune-mediated epilepsy can lead to early treatment and favorable therapeutic outcomes. Immunotherapy options for epilepsy include corticosteroids, intravenous immunoglobulins (IVIg), plasmapheresis, and steroid-sparing drugs [25]. The etiology of epilepsy remains unknown for some patients. Due to differences in medical conditions and economic levels between countries, it is impossible to make an accurate diagnosis of the etiology of some patients with epilepsy.

Metabolic epilepsy is caused by inherited metabolic disorders (IMD), and epilepsy or seizures are common symptoms in patients with some IMD, including energy metabolism defects, amino acid metabolism disorder, complex molecule and organelle metabolism defects, and neurotransmitter disorders [41]. About 42 percent of the genes involved in epilepsy were also found to be associated with IMD by gene sequencing [42]. A few metabolic seizures are acquired, such as folic acid deficiency in the brain. Most patients with metabolic epilepsy can be found on routine laboratory or metabolic tests. Glucose transporter 1 (GLUT1) is encoded by the solute vector family 2A1 (SLC2A1) gene. There was an association between GLUT1 expression and immune cell infiltration. A reduction in GLUT1 can lead to impaired energy metabolism, which can lead to seizures. When GLUT1 was highly expressed, the number of memory B cells decreased [43]. The signs and symptoms of metabolic epilepsy can overlap with other disorders. Studies have shown that 18% of metabolic epilepsy is treated in a specific way. Therefore, early diagnosis and identification of metabolic causes of epilepsy are very important to its treatment of it.

The most common form of childhood seizures, febrile seizures (FS), is usually induced by fever [44]. Dube *et al*. showed that IL-1β, a pyrogenic PIC is critical for the underlying heat-induced hyperexcitability of FS [45], and plasma levels of proinflammatory and anti-inflammatory factors were measured in 55 children with FS. Plasma IL-1RA/IL-1β ratios significantly increased in patients with FS patients and were mostly associated with it [46]. Similarly, increased levels of white blood cells have been found in the cerebrospinal fluid of children with FS [47]. White interpretation plays a vital role in FS, according to these results. In addition, IL-1β can promote the development of epilepsy through the following mechanisms: effects on neuronal survival and transcription pathways, rapid action on receptor-gated ion channels, and long-term effects on selective gene families [48], which may be associated with the development of epilepsy after febrile convulsions. The gram-negative bacterial endotoxin, lipopolysaccharide, can induce the release of inflammatory factors in macrophages. Injection of lipopolysaccharide-induced inflammation in rats decreased the seizure threshold and increased susceptibility to epilepsy, resulting in increased cytokine levels (IL-1β or TNFα) in the brains of epileptic rats. At the same time, the number of astrocytes and microglia increased in the CA1 region of the hippocampus of epileptic rats [49, 50].

**Fig. (1):**
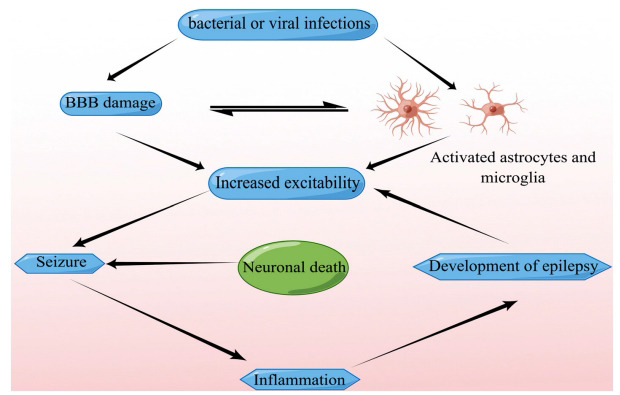
The pathophysiological cascades of inflammation in epilepsy. The pathological events caused by infection or immunity lead to the activation of microglia and astrocytes and the destruction of the blood-brain barrier (BBB). The inflammatory mediators released will enter the brain or blood through the damaged BBB, and then cause a series of pathophysiological results. Inflammation in the brain can cause seizures and cell death, which in turn creates a vicious cycle that leads to epilepsy. Drawing by Figdraw (www.figdraw.com).

Inflammation caused by infection may trigger seizures (Fig. **1**). Seizures can also trigger inflammation. Immunoreactive beta-amyloid and IL-1β levels were elevated in the human temporal lobe tissue of surgically resected patients with refractory epilepsy, and the number of microglia was three times higher in patients with epilepsy than in controls [51]. PICs are also induced in the brain by audiogenic and kindled seizures [52, 53]. Severe seizures during persistent epilepsy can cause neuronal death. Dead neurons further cause an inflammatory cascade, resulting in impairment of the BBB, which further promotes the development of epilepsy [30]. MRI was performed to study the temporal evolution of lesions in an epileptic rat model. At 2 h after status epilepticus (SE), BBB breakdown was observed only in the thalamus; it disappeared after 6 h [54]. Due to the activation of astrocytes and microglia, BBB dysfunction and neuronal death lead to the further development of epilepsy.

In addition, some autoimmune diseases can cause systemic inflammation, which may have an important association with epilepsy. Systemic lupus erythematosus (SLE) is a chronic autoimmune disease that affects multiple organs by activating the immune system. Studies have shown that the incidence of epilepsy in SLE patients with anticardiolipin antibodies is three times higher than that in patients without anticardiolipin antibodies [55]. Vasculitis caused by SLE lead to BBB damage. Interleukin-10 secreted by microglia increases the number of excitatory and inhibitory synapses and dendritic spins through the damaged BBB and may be a cause of seizures in patients with SLE [56]. Rheumatoid arthritis (RA) is an autoimmune inflammatory disease associated with rheumatoid factors and anti-cyclocitrullinate peptide antibodies, which involve inflammatory cytokines, such as TNFα, IL-1, and IL-6 [57]. Patients with temporal lobe epilepsy have higher levels of IL-1β, IL-6, and IL-1RA in their serum [58]. The incidence of epilepsy in patients with RA was 1.27-fold higher than that in control patients without RA. Patients with RA treated with nonsteroidal anti-inflammatory drugs have a reduced risk of epilepsy [59]. Psoriasis is an inflammatory condition of the skin produced by the immune system and manifests clinically primarily as erythematous plaques with silvery scales on the skin, including the nails and scalp [60]. Various immune cells are involved in the pathogenesis of psoriasis including the Th1 lymphocytes, CD4^+^ T cells, γδ T-cells, natural killer (NK) cells, and innate lymphoid cells (ILCs) [61]. Future studies on the association between this metabolic abnormality and seizures are needed. Inflammatory bowel disease (IBD) affects the digestive system, including ulcerative colitis (UC) and Crohn's disease (CD). T-helper (Th) 17 and regulatory T (Treg) cells are mainly involved in the immune regulation of IBD. The Th17/Treg is significantly increased in the peripheral blood of patients with IBD [62]. There is a significant increase in epilepsy risk among CD and UC patients, according to a retrospective study [63]. Type 1 diabetes mellitus (T1DM) is an autoimmune disease with beta-cell destruction. In a retrospective open cohort study, patients with T1DM had a three-fold increased risk of epilepsy compared to matched controls without diabetes [42, 64]. The antibodies associated with T1DM are glutamic acid decarboxylase (GAD) antibodies; however, not all T1DM patients with GAD antibodies have epilepsy. The probability of occurrence of TIDM increases only when the level of GAD antibody reaches a certain level [65]. GAD is mainly expressed in the CNS and is responsible for the synthesis of the major inhibitory neurotransmitter γ-aminobutyric acid (GABA). Therefore, anti-GAD antibodies may influence the occurrence of epilepsy by affecting GABA synthesis and GABA-receptor binding [65]. The association between this metabolic abnormality and seizures needs to be further studied in the future. By studying the relationship between epilepsy patients and 12 autoimmune disorders, Ganelin *et al.,* found that patients with psoriasis were about twice as likely to develop epilepsy as the control group [66]. Therefore, inflammation caused by certain infectious and immune factors can cause neuronal death, damage to the blood-brain barrier, release of proinflammatory factors by astrocytes and microglia to induce epilepsy, and seizures can also cause neuronal death and aggravate the inflammatory response. Therefore, it may be possible to prevent or limit the occurrence of epilepsy by controlling inflammation in the brain and targeting inflammatory mediators.

## 4 CURRENT TREATMENTS FOR EPILEPSY

Presently, the preferred treatment for epilepsy is antiepileptic drugs, and the existing antiepileptic drugs mainly prevent the occurrence of epilepsy through different mechanisms, including ion channel blockers, GABA enhancers, and glutamate blockers. The classification of antiepileptic agents according to different mechanisms of action is presented in Table **1**.

Voltage-gated ion channels, especially sodium, calcium, potassium, and hyperpolarization-activated cyclic nucleotide-gated (HCN) channels, are mainly associated with epileptic seizures. Blocking voltage-gated sodium channels is the most common mechanism of action among currently available antiepileptic drugs. The normal resting neuronal membrane potential is extracellular positive and intracellular negative. Usually, inhibition processes mediated by an inward chlorine or outward potassium current hyperpolarize the membrane, and excitation processes mediated by an inward sodium or calcium current depolarize the membrane [67].

Sodium channel blockers (phenytoin, carbamazepine, oxazepine) suppress seizures by reducing the influx of sodium [68-70]. Lacosamide can slowly inactivate voltage-gated sodium channels [71]. Currently, calcium channels associated with the α1 subunit mainly include L-, N-, P-, Q-, and T-type channels. Topiramate can block L-type calcium current [72]. Zonisamide (ZNS) is a third-generation antiepileptic drug that inhibits T-type calcium currents [73]. Lamotrigine acts mainly by blocking voltage-dependent sodium channels. In addition, it can modulate calcium channels and HCN channels [74]. GABA is an inhibitory neurotransmitter in the brain, essential for maintaining the balance between neuronal excitation and inhibition. It can transmit information from one neuron to another and block additional electrical activities [75]. GABA plays an inhibitory role in neurons by binding to membrane-bound receptors and acts mainly on two types of receptors: ionic receptors of ligand-gated ion channels (GABA_A_ and GABA_C_ receptors) and metabolic receptors of G-protein-coupled receptors (GABA_B_ receptors) [76, 77]. When GABA binds to GABA_A_ receptors, it increases cell membrane permeability to chloride ions, generating inhibitory postsynaptic potentials, leading to membrane hyperpolarization, and making it more difficult for neurons to reach action potentials. The binding of GABA to GABA_B_ receptors inhibits calcium channels or inactivates potassium channels. A primary goal of therapies targeting GABA-mediated neuronal inhibition is to restore equilibrium between excitation and inhibition [75]. Glutamate (Glu) is a main excitatory neurotransmitter in the brain. Glutamate promotes the influx of sodium and calcium ions and the outflow of potassium ions by binding to glutamate receptors. Glutamate receptors are mainly divided into ionic and metabolic glutamate receptors. The ionic glutamate receptors are divided into three types: N-methyl-D-aspartate (NMDA), α-amino-3-hydroxy-5-methyl-4-isoazole-propionic acid (AMPA), and 2-carboxy-3-carboxymethyl-4-isopropenylpyrrolidine (kainate) receptors [78]. Topiramate exerts its antiepileptic effects through various mechanisms, including ion channel blocking, GABA inhibition, and anti-glutamate effect [68]. Diazepam and lorazepam increase the frequency of opening the chloride channel of the GABA_A_ receptor [79]. In addition, levetiracetam exerts antiepileptic effects by interacting with the synaptic vesicle protein SVA2, which is suitable for focal and secondary tonic-clonic seizures and absence seizures [80, 81].

Currently, known antiepileptic drugs mainly control the symptoms of epilepsy and do not effectively protect the brain from cell death caused by epileptic seizures and prevent the future development of chronic epilepsy. Traditional antiepileptic drugs can cause a variety of adverse reactions, which can be divided into two kinds: early adverse reactions (cutaneous adverse reactions, somnolence, dizziness, gastrointestinal events) and late adverse reactions (psychotic episodes, behavioral problems, depression, impaired cognition, osteoporosis). Among them, the most common is a cutaneous adverse reaction. Aromatic anticonvulsants such as phenytoin (PHT), phenobarbital, and carbamazepine (CBZ) are the most common drugs that cause skin adverse reactions. Skin adverse reactions caused by antiepileptic drugs mainly include generalized morbilliform rashes and urticaria, which disappear in a few days. Severe skin adverse reactions can be life-threatening, including drug reactions with eosinophilia and systemic symptoms (DRESS), Stevens-Johnson syndrome (SJS), and toxic epidermal necrolysis (TEN). The adverse skin reaction caused by antiepileptic drugs may be anaphylactic hypersensitivity, and this allergic reaction is related to immune response [74]. The covalent adduct formed by the irreversibly binding of AEDs active metabolites to cellular proteins can bind to T cells as haptens and trigger an immune response [85]. Clinical studies have shown that PHT, CBZ, and valproic acid (VPA) inhibit immune activity, reduce the ratio of CD4^+^ to CD8^+^, and change serum concentrations of IgA, IgG, and IgM [80]. Epoxide hydroxylase detoxifies the metabolites of aromatic amine anticonvulsants, and the absence or abnormality of epoxide hydroxylase leads to the accumulation of toxic metabolites that trigger the immune response involved in DRESS (Table **1**) [81-87].

Therefore, new therapeutic targets and drugs need to be found. Inflammation can promote the occurrence and development of epilepsy by affecting the function of the BBB and the activation of astrocytes and microglia. Therefore, regulating the inflammatory response in the brain and targeting inflammatory mediators may be effective therapeutic strategies to prevent or limit the occurrence of epilepsy in the nervous system. Anti-inflammatory therapy given during the incubation period shortly after nervous system injury (before epileptic seizures) may reduce the effects of inflammation on the brain after injury, reduce the release of PICs and reduce the risk of epileptic seizures. Similarly, anti-inflammatory therapy given after epileptic seizures may reduce the inflammatory response to neuronal death caused by epileptic seizures and prevent further development of epilepsy. Treatments for epilepsy caused by autoimmune encephalitis include adrenocorticotropic hormone, plasmapheresis, glucocorticoids, monoclonal antibodies, and immunoglobulins. Due to the lack of randomized controlled trials evaluating the efficacy of immunotherapy for autoimmune epilepsy. Current immunotherapy recommendations are based on case series and clinical experience [73, 86]. Therefore, antiepileptic drugs are still the first choice for routine treatment of epilepsy caused by other causes.

The JAK-STAT signaling pathway is one of the key factors that promote neuroinflammation in neurodegenerative diseases, which has been proved by its multiple functions in neurology disease. Accumulation of misfolded synuclein and loss of dopamine neurons in the brain are important features of Parkinson's disease (PD). The JAK1/2 inhibitor AZD1480 inhibited inflammatory gene expression in microglia and macrophages by reducing STAT1 and STAT3 activation, preventing the degeneration of dopamine neurons *in vivo* [88]. The deposition of beta-amyloid (Aβ) protein in senile plaques is the primary pathology associated with Alzheimer's disease(AD). Studies have shown that nicotinic acetylcholine receptors can reduce Aβ neurotoxicity through JAK2-STAT3 activation and its derivatives inhibit the neurotoxicity of Aβ by activating the JAK2-STAT3 pathway and preserving cholinergic function [89, 90]. Multiple Sclerosis (MS) is an inflammatory, demyelinating disease of the central nervous system (CNS). Studies have shown that the activation of the JAK-STAT pathway can promote the survival of oligodendrocytes. This promotes the production of myelin and myelin proteins, enhancing myelin regeneration. SOCS3 can inhibit the JAK pathway and reverse this effect, confirming the above assumption. This effect can be used therapeutically to enhance myelin regeneration in the future [91]. Huntington's disease(HD)is a neurological disorder characterized by involuntary movements (chorea) and cognitive changes. SOCS3 significantly inhibited The JAK-STAT3 pathway in a nonhuman primate lentiviral vector-based model of HD, prevented astrocyte reactivity, and decreased microglial activation [92].

## 5 THE JAK-STAT SIGNALING PATHWAY

The cytokine receptor activates the JAK-STAT pathway through multiple combinations of JAK and STAT family members. Non-covalent binding of JAK to the cytokine receptor mediates tyrosine phosphorylation of the receptor, followed by STAT phosphorylation to form homodimers, isodimers, or tetramers. They then move from the cytoplasm to the nucleus, where they act as transcription factors regulating gene expression [93, 94]. When type I and type II cytokines bind to their homologous receptors, JAK-STAT signaling is activated. Each receptor is composed of multiple subunits, and the specificity of type I and type II cytokine binding to JAK or STAT depends on the type of subunit of the homologous receptor (Table **2**). JAKs and STATs are the components of JAK-STAT signaling. In mammals, the JAK family consists of four members: JAK1, JAK2, JAK3, and Tyk2. The STAT family currently consists of seven members: STAT1, STAT2, STAT3, STAT4, STAT5a, STAT5b, and STAT6.

### 5.1 The JAK Family

**Fig. (2):**
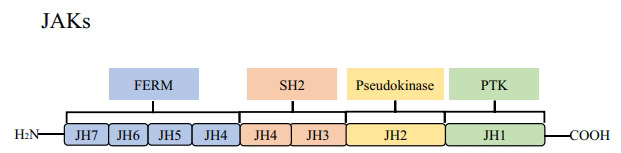
Diagram of JAK structural domains. There are four functional domains and JAK homology domains (JH). Four functional domains: kinase, pseudokinase, four-point-one protein, Ezrin, Radixin, and Moesin domain (FERM), and Src homology 2 (SH2) domains.

There are four members of the JAK family: JAK1, JAK2, JAK3, and Tyk2. JAK1, JAK2, and Tyr2 are expressed in almost all tissues [95-97]. JAK3 is preferentially expressed in the hematopoietic cells, lymphocytes, endothelial cells, and vascular smooth muscle cells [98-100]. JAKs contain four domains (Fig. **2**): the FERM domain (F for 4.1 protein, E for ezrin, R for radixin, and M for moesin), which consists of 298 amino acids, is the connectome of the membrane cytoskeleton [101]. The Src homology 2 (SH2) domain interacts with tyrosine phosphorylation sites to recruit statins to receptor complexes [102]. The pseudokinase domain is a kinase-like domain (KL) with catalytic activity [103]. Protein tyrosine kinases (PTK) exist mainly in multicellular organisms and are particularly important in cell differentiation and processes related to specific cell functions [104]. There are seven JAK homology domains (JH) in the JAK structure.

The JAK1 human gene is located on chromosome 1p31.3 and JAK2 on chromosome 9p24 [105]. JAK3 is located at 19p13.1, and Tyk2 is located at 19p13.2 [96, 106]. JAK1 is approximately 135 kDa, JAK2 is 130 kDa, JAK3 is 120 kDa, and Tyk2 is 140 kDa [107]. The activation of JAK2, TYK2, and STAT4 is critical to the differentiation of Th1 cells, while the activation of JAK1/3 and STAT6 is critical for the Th2 cell differentiation [108]. JAK1 is activated by cytokine receptors with a γc receptor subunit and receptors with a gp130 subunit. Additionally, type II cytokine receptors can effectively activate STAT1 [109]. JAK2 is a non-receptor tyrosine kinase that promotes cell growth and proliferation. It can transmit a variety of cytokine signals and participates in inflammatory signal transduction [110]. JAK2 can also be phosphorylated by cytokine receptors, with the βc receptor and type II cytokine receptors [111]. JAK3 is mainly related to immune function and participates in the formation of various cytokines such as IL-2, IL-4, IL-7, and IL-9 [112]. JAK3 primarily binds to receptors with γ receptor chains, including IL-2, IL-4, IL-7, IL-9, IL-15, and IL-21 [113]. Tyk2 plays a pivotal role in innate and acquired immunity in humans and is activated mainly by type II cytokines [109].

### 5.2 The STAT Family

**Fig. (3):**
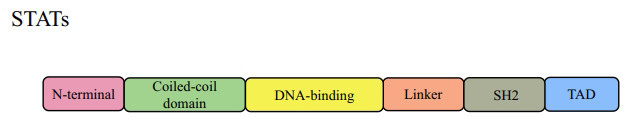
Schematic of STAT structural domains. STAT proteins contain six domains: an N-terminal domain (ND), the coiled-coil domain (CCD), the DNA-binding domain (DBD) located amid the protein, the linker domain (LD), the SH2 domain and the transcriptional activation domain (TAD).

There are seven members of the STAT family: STAT1-4, STAT5a-b, and STAT6. STATs have six different domains (Fig. **3**): an N-terminal domain (ND), a coiled-coil domain (CCD), a DNA-binding domain (DBD) located within the protein, a linker domain (LD), an SH2 domain, and a transcriptional activation domain (TAD). The ND-mediated oligomerization enables Stat5 to extend its range of target sites considerably by utilizing nonconsensus-binding sites [114]. The CCD, necessary for the interaction between STAT3 and the IL-6 receptor, consists of four antiparallel helices [115]. The DBD, located between amino acids 400 and 500, forms a complex with DNA and STAT proteins to bind the regulatory sequence of the target gene [109]. The linking domain, as an independent transactivation domain, connects the DBD to the SH2 domain [114, 116]. The SH2 domain interacts with a variety of proteins to influence multiple signaling pathways.

The SH2 domain binds STAT to the receptor complex by interacting with its tyrosine phosphorylation site [102]. Tyrosine and serine phosphorylation sites are present in the TAD that is required for STAT activation [117].

STAT1 (composed of two isomers STAT1α (91 kD) and STAT1β (84 kD)) [116] is mainly activated by IFN and has the following biological functions: inhibition of cell growth, regulation of cell differentiation, promotion of cell apoptosis, inhibition of tumor occurrence, and regulation of the immune system [109]. In humans, STAT2 is located on chromosome 12 [101], and cannot bind to DNA directly but can bind to the DNA-binding protein IRF-9 by forming a dimer with STAT1α or STAT1β [116]. The biological functions of STAT2 include antiviral action, immunomodulatory activity, and tumorigenesis [109]. STAT2 is activated by IFN-α and IFN-β. STAT3 consists of two alternately spliced isomers: full-length STAT3α and truncated STAT3β [118]. Compared with STAT1 and STAT2, STAT3 is not only responsible for regulating cell growth, differentiation, apoptosis, and immunity but is also involved in the regulation of cancer stem cells [109]. STAT3 is activated by IL-6 family members, IL-10 family members, IL-21, IL-27, granulocyte colony-stimulating factor (G-CSF), leptin, and IFN-Is [119, 120]. Similar to STAT1, STAT4 contains two types of clipped transcripts: STAT4α and STAT4β. STAT4 is activated by IL-12, type I interferon (IFN-I), IL23, IL27, and IL35, *etc*. STAT4 can increase the Th1 cell differentiation, cytotoxicity, and IFNγ production by immune cells, and regulate tumor cell migration and proliferation [121]. The transcription factor STAT4 is activated by IFN-I, IL-12, and IL-23 [122, 123]. Unlike other STAT members, STAT5 comprises STAT5a and STAT5b. STAT5a can form dimers and tetramers that bind to DNA, whereas STAT5b forms dimers [124]. STAT5 protein is activated by a variety of cytokines and growth factors, including prolactin, growth hormone (GH), IL-2, IL-7, IL-9, IL-15, IL-3, IL-5, erythropoietin, and thromboietin [105]. Recently, STAT5 has been shown to have biological functions in cell growth, differentiation apoptosis, and tumor immune regulation [109]. The human STAT6 gene is located on chromosome 12q13-q14. STAT6 signaling is primarily activated by IL-4 and the highly related cytokine, IL-13. STAT6 has several biological functions in the immune system. When STAT6 is altered, it can lead to allergic reactions, tumors, and autoimmune diseases [109, 125]. The cytokines that activate STAT6 include IL-4 and IL-13 [126].

### 5.3 Activation of JAK-STAT Signaling Pathways

**Fig. (4):**
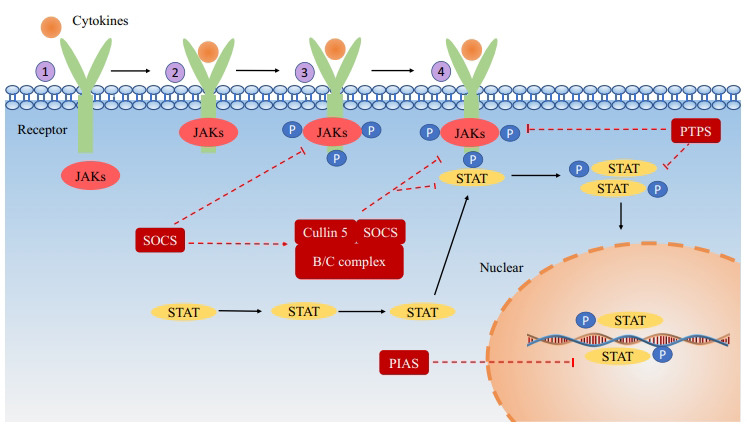
The activation and negative regulation of the JAK-STAT pathway. Negative regulation is indicated by red dotted arrows, while activation is indicated by black arrows. JAK-STAT activation: **①** The binding of cytokines to receptors leads to dimerization of receptor molecules, activating JAKs. **②** The STAT protein is attracted to the docking sites created by these phosphorylated tyrosine sites. **③** As a result of being phosphorylated and activated, STATs form homodimers or heterodimers. **④** A STAT-STAT dimer translocates to the nucleus and controls gene expression. JAK-STAT inhibition: The JAK-STAT pathway is negatively regulated by three different types of proteins: suppressor of cytokine signaling (SOCS), protein inhibitor of activated STAT (PIAS), and protein tyrosine phosphatases (PTPs). Three ways are available to the SOCS family for negatively regulating the JAK/STAT pathway: (1) The SOCS protein binds to the phosphotyrosine on the receptor to prevent STAT from being recruited. (2) SOCS inhibits JAK activity by directly binding JAK or its receptor. (3) SOCS forms a B/C-cullin5 complex that degrades JAK or STAT bound to the SOCS protein through polyubiquitination. PIAS inhibits STAT binding to DNA by interacting with STAT dimers. PTPs can dephosphorylate the JAK-binding substrate and dephosphorylate and inactivate the STAT dimer.

At present, there are two main activation methods for the JAK-STAT pathway: canonical pathway and noncanonical pathway. According to the canonical JAK-STAT pathway, receptor dimerization of receptor-associated JAK kinases through binding of ligands (*e.g*., cytokines) to transmembrane receptors leads to phosphorylation of JAK to form a STATs docking site (Fig. **4**). JAK phosphorylates STAT at this docking site. STAT separates from the receptor *via* SH2-domain phosphotyrosine interactions and forms homodimers or heterodimers. Dimers bind to the promoters of target genes and regulate transcription.

Unlike typical signaling patterns, various STAT3 target genes can be expressed in response to unphosphorylated STAT3. In an atypical pattern, a portion of unphosphorylated STAT is localized to the nucleus of heterochromatin protein-1 (HP1)-associated heterochromatin. The unphosphorylated STAT associated with heterochromatin is critical for HP1 localization and heterochromatin stability. The activation of STAT by phosphorylation results in the diffusion of STAT from heterochromatin, which leads to HP1 replacement and heterochromatin instability [127, 128]. Drosophila has confirmed the existence of a non-classical pathway, and unphosphorylated mammalian STAT proteins affect gene transcription through a mechanism different from that of phosphorylated STAT [128-130].

### 5.4 Negative Regulators of JAK-STAT Signaling Pathways

The JAK-STAT pathway is regulated by a variety of negative regulators. To date, there are three main negative regulatory factors: suppressor of cytokine signaling (SOCS), protein inhibitor of activated STAT (PIAS), and protein tyrosine phosphatases (PTPs) (Fig. **4**).

The SOCS, together with the cytokine-induced SH2 domain protein (CIS), form a family of intracellular proteins. There are eight main members of this intracellular protein family: SOCS1-7 and CIS. Activated STAT forms a dimer and enters the nucleus where it induces SOCS expression. The SOCS inhibits the JAK-STAT signaling pathway by binding to phosphorylated JAK. The SOCS protein consists of an N-terminal region of varying length and sequence, a central Src homology 2 (SH2) domain, and a carboxy-terminal 40-amino acid module, known as the “SOCS box” [131]. Negative regulation of SOCS is mainly carried out in the following three ways: (1) SOCS binds to phosphotyrosine on the receptor to prevent STAT from attaching to the receptor [132]. (2) SOCS specifically binds JAK or its receptor to inhibit the JAK kinase activity [109]. (3) The SOCS box, cullin5 scaffold protein, and elongation protein B/C complex bind to form an elongation protein-cullin-SOCS3 E3 ubiquitin-linked enzyme complex [133]. This complex degrades SOC-bound JAKs and STATs *via* polyubiquitination [109].

The mammalian PIAS protein family mainly consists of four members: PIAS1, PIAS3, PIASx (PIASxα and PIASβ), and PIASy. A protein named GBP (Gu/Rh-II binding protein) is closely associated with PIAS1, which lacks the NH2 terminal 9 amino acid residue compared with PIAS1, so PIAS1 may be a complete version of GBP [134]. Studies have shown that PIAS1 and PIAS3 specifically inhibit STAT1 and STA3 expression, respectively. The DNA-binding activity of STAT is blocked by PIAS1 and PIAS3 to inhibit STAT-mediated gene activation [135, 136], and PIASx and PIASy interact with STAT4 and STAT1, respectively. PIASx and PIASy inhibit transcription without affecting STAT DNA-binding activity [137, 138]. The PTPs family can be divided into three categories according to the groups in the catalytic center: Cys-based phosphatases (classes I, II, and III), Asp-based phosphatases, and His-based phosphatases [139]. The PTPs can dephosphorylate the JAK-binding substrate and dephosphorylate and inactivate the STAT dimer through the SH domain, thus inhibiting the JAK-STAT signaling pathway [140, 141].

In addition, STAT-interacting LIM proteins (SLIM) and microRNAs can modulate the JAK-STAT pathway by inhibiting STAT4. The SLIM is a nuclear ubiquitin E3 ligase that inhibits the JAK-STAT pathway by promoting STAT4 degradation and dephosphorylation [142]. A number of miRNAs have been shown to interfere with IL12-STAT4 signaling [121]. In primary human NK cells, miRNA132, -212, and -200 up-regulation negatively regulates the IL-12 signaling pathway by reducing STAT4 expression [143].

The JAK-STAT3 pathway plays a key role in the control of astrocyte reactivity. The STAT3 protein is involved in regulating the production of cytokines and chemokines during inflammation in astrocytes. Astrocytic with the treatment of high glucose upregulated the expression of TNF-α, IL-6, IL-1β, and IL-4, and this process can be blocked by STAT3 inhibitors [144]. Astrocytes also secrete a variety of trophic factors including brain-derived neurotrophic factor (BDNF), glial cell line-derived neurotrophic factor, and leukemia suppressor (LIF). Studies have shown that certain cytokines and nutritional factors activate the JAK-STAT pathway to promote neuroprotection [145]. The activation of STAT through signal transduction of the LIF receptor can enhance the survival rate of oligodendrocytes, which is an effective treatment strategy for demyelinating disease [91]. In astrocytes, STAT3 reduces reactive oxygen species production and increases levels of the antioxidant glutathione to reduce oxidative stress levels [146]. In addition, STAT3 can regulate autophagy by regulating the expression of autophagy-related genes [147]. Microglia, like astrocytes, can also release a variety of nutritional factors, such as BDNF, nerve growth factor, hepatocyte growth factor, epidermal growth factor, and others. BDNF can affect the JAK-STAT pathway to regulate the expression of GABA receptors. Pathological changes can induce the activation of microglia. Activated microglia have two phenotypes: M1 and M2. M1 phenotype activation is associated with proinflammatory cytokines and M2 phenotype activation is associated with anti-inflammatory cytokines. The released cytokines may activate the JAK-STAT pathway by binding to the receptor [148]. Microglia are also associated with autophagy. TLR4-dependent autophagy might regulate microglial polarization and could induce ischemic white matter damage *via* the STAT1/6 pathway [149]. In addition, activated microglia can up-regulate the expression of MHC II, become antigen-presenting cells, and activate CD4^+^T cells to participate in the pathogenesis of PD [150].

## 6 THE JAK-STAT SIGNALING PATHWAY IN EPILEPSY

A growing body of research suggests that the JAK-STAT pathway may be involved in epilepsy. After analyzing differential gene expression in rat hippocampal cells in a pilocarpine model of epilepsy, Okamoto *et al.* found activation of JAK-STAT signaling pathways during epileptic seizures. The expression profiles of JAK3 and STAT3 were up-regulated in the hippocampus of pilocarpine-treated rats at different times during epileptic onset compared with the control group. The finding reveals dynamic molecular changes occurring in the hippocampus, which may provide a reference for future research on alternative therapeutic strategies for preventing epilepsy after acquired brain injury [151]. Similarly, pSTAT3 in the hippocampus was significantly increased at 24 h and at different time points in the hippocampus, cortex, and cerebellum of mice with epileptic persistence. STAT3 increased by 68% at 24 hours. In the chronic phase, it increased again, reaching 55%, and the STAT3 levels in the cortex increased by 63% after 24 hours [152].

The JAK-STAT pathway may affect the occurrence and development of epilepsy by affecting neuronal excitability, neuronal migration, neuronal synchronization, glial cell activation, and synaptic plasticity.

To investigate the effect of peripheral inflammation on seizure susceptibility and neuronal excitability through immune signaling, 2,4,6-trinitrobenzene sulfonic acid (TNBS) was injected into the colons of rats to initiate a model of T-helper 1 cell-mediated inflammatory bowel disease. Riazi *et al.* examined brain excitability by administration of pentatetrazole to induce clonic seizures in mice. Following the application of 4-aminopyridine to hippocampal slices from TNBS-treated rats, spontaneous interictal burst firing was increased, suggesting increased intrinsic excitability [153]. The release of inflammatory mediators occurs as a result of the inflammatory response. In the brain, IL-1β functions mainly by increasing NMDA receptor function and enhancing the concentration of extracellular glutamate [154]. In addition, IL-1β inhibits glutamate reuptake, which results in increased excitability in neurons and increases extracellular glutamate levels [155, 156]. The increased neuronal excitability and astrocytic activation induced by IL-1β may lead to increased glutamatergic transmission, resulting in seizure activity [157]. IL-1β has also been reported to inhibit GABA-mediated Cl− fluxes, potentially causing hyperexcitability by reducing inhibitory transmission [158]. Lipopolysaccharide (LPS)-induced release of proinflammatory mediators and microglial proliferation and migration rates are also significantly increased, accompanied by activation of the JAK-STAT pathway [159]. Hypogonadotropic hypogonadism is caused by impaired hypothalamic gonadotropin-releasing hormone (GnRH) neuron migration or GnRH regulation. After the knockdown or scrambling of the nasal embryonic luteinizing hormone-releasing factor (NELF) gene in the GnRH neurons, RNA was extracted and cDNA assays were carried out. Ko *et al.* found that the expression of transcription factors and cell migration genes in GnRH neurons was frequently altered. The above results were found to be related to the regulation of the JAK-STAT signaling pathway by NELF in GnRH neurons [160]. Known as a hyper synchronized state of neurons, epilepsy is a functional brain disorder associated with the excessive synchronization of large populations of neurons. GABA is the major neurotransmitter that inhibits the brain. At present, GABA receptors are mainly divided into GABA_A_, GABA_B,_ and GABA_C_. GABA_A_ and GABA_C_ are ionotropic receptors; GABA_B_ is a metabotropic receptor. GABA_A_ receptors are composed of multiple subunit subtypes (α1-6, β1-3, γ1-3, δ, ε, θ, π, and ρ1-3). The GABA_A_ receptor (GABA_A_R) is the major inhibitory neurotransmitter receptor in the brain. Studies have shown that BDNF regulates the JAK-STAT pathway to reduce the expression of the GABA_A_Rα1 subunit associated with epilepsy [161]. Gabaergic inhibition has traditionally been seen as the main mechanism for balancing glutamatergic excitation and preventing the firing of hypersynchronized neurons. The JAK-STAT pathway activation and reduced GABA_A_Rα1 subunit levels result in decreased inhibition and increased excitation, causing intense, hypersynchronous neuronal activity. Recordings of electroencephalography (EEG) during such episodes demonstrate ictal (seizure) discharges of high amplitude [162].

The glial cells (astrocytes, microglia, and oligodendrocytes) are involved in the pathophysiology of a variety of neurological diseases. To date, clinical and animal studies have revealed that astrocyte dysfunction can cause hyperexcitability, neurotoxicity, and seizures. The European Commission of the ILAE also suggested that glial cells and inflammation may influence seizure development and progression [163]. Neuroinflammation causes neuronal hyperexcitability and seizures, which promote proinflammatory cytokine production, triggering inflammatory mediator cascades downstream. Thus, a malignant positive feedback loop is formed between seizures and inflammatory mediators. In a kainic acid (KA) fire model, 7 days after seizure induction, STAT3 increased the levels of IL-1β, IL-6, and TNF-α, as well as the activation of astrocytes and microglia in the hippocampus of epileptic rats. The use of the STAT3 inhibitor AG490 inhibited the activation of glial cells and inflammatory responses [164]. Similarly, another study showed that 1 day after KA-induced seizures in mice, STAT3 activation caused microglial activation and neuroinflammation associated with monocyte infiltration [165].

Synaptic plasticity refers to the ability of neurons to modify synaptic transmission, and in the mammalian brain, long-term potentiation (LTP) and long-term inhibition (LTD) are two forms of persistent synaptic plasticityEvidence suggests that the JAK-STAT pathway affects synaptic plasticity *via* the AMPA and NMDA receptors [166]. Administration of the JAK inhibitor AG490 blocked the induction of NMDAR-LTD in rat hippocampal sections. In organic-type sections transfected with JAK2 short hairpin RNA(shRNA) and STAT3 shRNA, CA1 cells in patch-clamp recording sections failed to induce LTD. This suggests that JAK phosphorylation and NMDA receptor activation promote synaptic stimulation. Therefore, LTD is regulated by the JAK-STAT pathway, and the cytoplasmic activity of STAT3 plays a crucial role in synaptic plasticity [167]. Lithium-pilocarpine was used to induce seizures in 3-week-old rats, and neuronal death was observed on the first day after the seizure. However, there was no impairment of synaptic plasticity at that time. A decrease in LTP caused by the weakening of NMDA receptor-dependent signaling was observed 3 days later. The negative effects of status epilepticus on plasticity increased after 1 week, accompanied by astrocytosis. According to these results, synaptic plasticity is impaired by a disorder of neuron-astrocyte interactions [168]. Leite *et al.* found that synaptic changes in experimental models of epilepsy involving dendritic spines and postsynaptic density (PSD) during LTP were accompanied by changes in spine morphology, an increase in the proportion of perforated PSD synapses, and the formation of multiple spinal buttons from the same dendrite [169]. These results suggest that JAK-STAT-affected NMDA receptor-LTD as well as disruption of neuron-astrocyte interactions contribute to synaptic plasticity damage.

Some antibody-mediated encephalitis, such as those associated with autoantibodies to GABAR, AMPAR, AMPAR, or Leucine-rich glioma-inactivated 1 (LGI1), may cause seizures accompanied by synaptic dysfunction, impaired synaptic plasticity, and hyperexcitability. GABAR-mediated inhibitory postsynaptic currents (IPSCs) and miniature IPSCs can block glutamatergic transmission. When hippocampal neurons were treated with serum from patients with GABA encephalitis, the number of synaptic GABA_A_R clusters were significantly reduced. The frequency of miniature IPSCs was also reduced in neurons treated with patient serum, which ultimately caused abnormal neuronal firing [170]. After injecting mice with cerebrospinal fluid antibodies from patients with NMDAR encephalitis. There was a significant reduction in NMDAR levels in the hippocampus, which was accompanied by severe impairments in learning and memory as well as a reduction in LTP [171, 172]. In rat brains, NMDAR antibodies can affect synaptic plasticity in neurons, resulting in decreased inhibitory synaptic density in excitatory hippocampal neurons [173]. When injected intraventricularly into mice with a patient's NMDAR antibody, Wright *et al.* found that mice were more sensitive to PTZ-induced seizures [174]. Like NMDARs, The AMPAR is a subunit-based excitatory glutamatergic receptor comprised of four GluA1-4 subunits. Antibodies against either GluA1 or GluA2 subunits cause AMPAR surface levels to be reduced in patients with anti-AMPAR encephalitis [175, 176]. The concentrations of AMPAR subunits GluA1 and GluA2 and NMDAR subunit NR2B were significantly reduced in a chronic temporal lobe epilepsy model [177, 178]. Borderline encephalitis with anti-LGI1 antibodies can also cause seizures. There is a trans-synaptic complex formed by LGI1 that includes presynaptic disintegrin and metalloproteinase domain-containing protein 23 (ADAM23) (which is associated with the potassium channels Kv1.1). When LGI1 patient-derived immunoglobulin G (IgG) antibody was injected into mice, a significant increase in evoked excitatory postsynaptic current (eEPSC), neuronal hyperexcitability, and increased glutamatergic transmission was observed by patch-clamp in hippocampal slices of mice, which may be regulated by the Kv1.1 potassium channels [179]. Analysis of synaptic plasticity in the CA1 region of the hippocampus revealed that LTP was severely impaired, which may be mediated by ineffective recruitment of postsynaptic AMPAR [180]. These results suggest that the LGI1 antibody can inhibit the interaction of LGI1 with ADAM22 and ADAM23, thereby affecting Kv1.1 potassium channels and AMPAR.

Although autoimmune-related encephalitis antibodies by influencing the excitability of neurons and synaptic plasticity-induced seizures, existing only in the study reported a JAK-STAT pathway can adjust GABAR, on the other hand, although the JAK-STAT pathway related to the immune, but is involved in regulating the immune encephalitis and its related receptor remains to be further research.

## 7 POTENTIAL INHIBITORS OF THE JAK-STAT PATHWAY AS A NOVEL TREATMENT STRATEGY FOR EPILEPSY TREATMENT

### 7.1 JAK Inhibition

JAK inhibitors are classified as first- and second-generation and are currently used primarily for a variety of indications, including arthritis, allergy, graft-*versus*-host disease (GVHD), IBD, psoriasis, and lupus. First-generation JAK inhibitors inhibit more than one type of JAK, whereas second-generation JAK inhibitors can selectively inhibit a single JAK to improve efficacy and reduce side effects.

#### 7.1.1 First-generation

Tofacitinib mainly inhibits JAK1 and JAK3 and inhibits JAK2 to some extent. Besides being used in psoriasis [181, 182], tofacitinib is also used in the treatment of alopecia, vitiligo, psoriatic arthritis, and UC [183-185]. In addition, tofacitinib improves cardiometabolic and immunological parameters associated with premature atherosclerosis in SLE [186]. Recent studies have shown that tofacitinib reduces inflammation during COVID-19 and significantly reduces mortality from COVID-19 [187, 188]. The most common adverse reactions to lovastatin are infections of the respiratory and urinary systems and skin-related infections, such as shingles. Patients treated with tofacitinib had decreased hematologic hemoglobin levels, and reduced neutrophil and lymphocyte counts. Some patients develop urticaria, angioedema, and rash [183]. Ruxolitinib competitively inhibits ATP-binding catalytic sites on JAK1 and JAK2 [189]. Ruxolitinib is a medication used to manage refractory acute graft-*versus*-host diseases. The most common adverse events observed with ruxolitinib include ecchymosis, headache, dizziness, anemia, and thrombocytopenia [7]. Baricitinib further affects the signaling of a variety of cytokines by inhibiting JAK1 and JAK2 [190]. Existing research suggests that barisinib improved psoriasis, specific dermatitis, alopecia areata, and RA [182, 191-193]. In addition, baricitinib in combination with remdesivir increased the outcome of treatment in patients with COVID-19 [188, 194]. Peficitinib is a JAK1, JAK2, JAK3, and Tyk2 (pan-JAK) inhibitor that is indicated for treating RA, CD, and UC [195, 196]. Oclacitinib is also a pan-JAK inhibitor shown to improve allergic dermatitis in dogs [197, 198].

#### 7.1.2 Second-generation

A selective JAK1 inhibitor, upadacitinib is approved for the treatment of moderate-to-severe RA, CD, and atopic dermatitis [199-202]. Upadacitinib is highly selective for JAK1 and very weak for other JAKS, so its use can reduce some of the adverse reactions. The JAK1 inhibitor filgotinib is the latest therapy approved for treating RA [203]. Fedratinib, a selective JAK2 inhibitor, was approved in the United States in 2019 for the treatment of intermediate-risk primary or secondary myelofibrosis. Common clinical side effects include diarrhea, dizziness, nausea, vomiting, and elevated levels of serum creatinine, lipase, amylase, and aminotransferase [204]. Decernotinib is a new jakinib that selectively blocks JAK3. Decernotinib is currently used as a JAK inhibitor in the treatment of RA and psoriasis. The most common adverse events were nausea, headache, diarrhea, upper respiratory tract infections, and hypercholesterolemia [182, 205]. Gandotinib is a potent and selective small-molecule inhibitor of JAK2. Recently, gandotinib has been shown to be safe and tolerated in phase I and II clinical trials in patients with primary myelofibrosis, polycytopenia, and primary thrombocytopenia [205, 206].

First-generation jakinibs block multiple JAKs, so first-generation jakinibs have more adverse reactions including infection, anemia, hyperlipidemia, *etc*. In addition, jakinibs also reduce creatinine clearance. Ruxolitinib has been shown to inhibit the expression of NLRP3 inflammasome and pSTAT3 proteins in neurons and microglia in middle cerebral artery occlusion (MCAO) mice, and to inhibit the expression of many proinflammatory factors, including TNF-α, IL-1β, IL-2 and IL-6 [207].

### 7.2 STAT Inhibition

As an important member of the JAK-STAT signaling pathway, The STAT gene has been shown to be a target for the treatment of several diseases. It blocks the phosphorylation and activation of STAT by blocking one or more JAK members, which may affect other signaling pathways. STAT inhibitors are more specific than JAK inhibitors and have fewer side effects. Recently, STAT3 and STAT5 inhibitors are the most widely studied inhibitors of the STAT family. Among the STAT3 inhibitors, small-molecule inhibitors and natural inhibitors are the most common. Therefore, we mainly introduced STAT3 and STAT5 inhibitors.

#### 7.2.1 Small-molecule STAT3 Inhibitors

Small STAT3 inhibitors are classified according to their chemical scaffolds, namely quinone derivatives, benzo [b] thiophene-1, 1-dioxygen derivatives, curcumin derivatives, quinoline derivatives, and salicylic acid derivatives [8].

Small-molecule STAT3 inhibitors containing quinone stents inhibit the expression of downstream target genes of STAT3 by inhibiting its DNA binding, phosphorylation, dimerization, and transcriptional activity of STAT3. STAT3 inhibitors containing the benzo [b] thiophene 1, 1-dioxide scaffold target mainly the SH2 domain, thus selectively inhibiting dimerization and nuclear translocation. The curcumin and derivatives of curcumin inhibit the activation of STAT3 by IFNα and IL-6 and the subsequent nuclear translocation of STAT3 [208]. Small-molecule STAT3 inhibitors based on salicylic acid scaffolds are being developed to target the SH2 domain. Quinoline derivatives are novel inhibitors of STAT3 dimerization that can selectively eliminate the DNA-binding activity of STAT3 [209].

#### 7.2.2 Natural STAT3 Inhibitors

Selective natural STAT3 inhibitors can be classified according to their chemical structure, including terpenoids, alkaloids, and other types.

There are three main types of natural terpene inhibitors: 1. Those that inhibit STAT3 phosphorylation by binding to the SH2 domain of STAT3. 2. Those that bind to the DBD of STAT3. 3. Inhibitors of JAK and STAT3 [8]. Berberine and homoharringtonine inhibit STAT3 activation in tumor cells [185, 186]. Other STAT3 inhibitors, such as galiellactone, can inhibit STAT3 binding to DNA. Ilamycin C can inhibit the phosphorylation of JAK2 and STAT3 [210-213].

#### 7.2.3 STAT5 Inhibitors

STAT5 inhibitors can inhibit any key stage of its activation and are currently used in the treatment of cancer, especially hematological tumors. There are three STAT5 inhibitors that interact and reduce its activity. The first inhibitor inhibits the tyrosine phosphorylation of STAT5. Pimozide is a potential STAT5 inhibitor that may be used to treat chronic myeloid leukemia (CML). By blocking tyrosine phosphorylation of STAT5, pimozide can reduce the expression of STAT5 target genes in KU812 and KU562 cells, reduce cell viability, and induce tumor cell apoptosis [214]. Another study demonstrated that pimozide effectively reduced STAT5 peripheral T-cell lymphoma (PTCL), an aggressive form of non-Hodgkin's lymphoma, and blocked STAT5 activation by binding to its SH2 domain [215]. The small non-peptide compound niacinamide attenuates STAT5 phosphorylation, thereby preventing its binding to DNA. The second class of STAT5 inhibitors, including BP-1-108, 13A, and AC-4-130, reduced STAT5 activation and target gene expression in different cells. The third type of inhibitor is a STAT5 transcriptional activity inhibitor that binds to brominated dopamine, a transcription regulator bromodomain containing 2 (BRD2). The JQ1 attenuates STAT5 transcriptional activity by inhibiting the STAT5-BRD2 interaction [216]. In addition, 21-mer decoy oligodeoxynucleotide (dODN) inhibits STAT5 activation by blocking its DNA-binding region [217].

However, the development of STAT inhibitors still faces several challenges. Owing to the high homology between STAT1 and STAT3, the use of STAT3 inhibitors may cause untargeted blocking of STAT1. In addition, in response to IL-6 stimulation, STAT3-deficient cells complete relevant signal transduction by activating STAT1 instead of STAT3 [218]. Thus, when STAT is specifically blocked, other members of the STAT family may compensate for this. The compensatory effect of STAT should be considered when developing new STAT inhibitors. Therefore, the development of STAT inhibitors with multiple targets may be a better choice. The main challenges currently facing the development of STAT inhibitors are bioavailability and selectivity issues. As STAT inhibitors are currently used in cancer treatment, the primary adverse effect is increased tumor cell survival [219].

## CONCLUSION AND FUTURE PERSPECTIVE

In this review, we discuss the composition, activation, and related positive and negative regulators of the JAK-STAT signaling pathway and introduce the possible mechanism underlying the effect of JAK-STAT on epilepsy from the perspectives of neuronal excitability alteration, neuronal migration, neuronal synchronization, activation of glial cells, synaptic plasticity and autoimmune encephalitis related receptors. Although there are few clinical and animal studies on JAK-STAT and epilepsy, this signaling pathway may be a potential target for epilepsy therapy in the future. However, there are some problems regarding this signaling pathway and epilepsy. First, although there are related JAK and STAT inhibitors in clinical practice, whether these drugs can be used directly in patients with epilepsy requires further clinical trials. Second, the role of the JAK-STAT pathway in the pathogenesis of epilepsy and whether the JAK-STAT pathway co-affects epilepsy with other signaling pathways have not been fully clarified. Third, the role of negative regulators in the JAK-STAT pathway in the treatment of epilepsy should be carefully evaluated. Fourth, the noncanonical pattern of JAK-STAT signaling regulates heterochromatin stability. Whether heterochromatin is related to the development of epilepsy requires further study. Fifth, whether JAK and STAT inhibitors interact with antiepileptic drugs and whether the inhibitors reduce or increase the concentration of antiepileptic drugs should be evaluated. Therefore, clinicians should pay close attention to the blood concentrations of antiepileptic drugs in patients with epilepsy when using JAK-STAT inhibitors. Sixth, the optimal duration of inhibitor use also needs to be determined in subsequent clinical studies, and whether inhibitors should be continued after the inflammatory response disappears. Seventh, whether JAK inhibitors are further involved in the development and progression of epilepsy by affecting neurons and synapses need further investigation. Lastly, owing to the large number of members of the JAK and STAT families, the effect of the selection of different inhibitors on patients with different types of epilepsy needs to be verified by further clinical trials. Antiepileptic drugs have indications, and the selection of appropriate inhibitors for different types of epilepsy may reduce adverse reactions.

Therefore, we consider the JAK-STAT pathway to be a potential target for therapeutic intervention in epilepsy. Further in-depth analyses of the regulatory role of the JAK-STAT pathway in epilepsy will be a significant direction of future epilepsy research. Furthermore, a greater focus should also be put on the role of negative regulators of the JAK-STAT pathway in treating epilepsy.

## Figures and Tables

**Table 1:** Current drug treatments for epilepsy.

**Drugs**	**Mechanism**	**Clinical Use**	**Side Effects**	**References**
Phenytoin	Block sodium channels	Partial and tonic-clonic seizures	Vertigo, hirsutism, Rash	[68]
Carbamazepine	Block sodium channels	Partial and tonic-clonic seizures	Hyponatremia, dizziness, somnolence, nausea, headache, diplopia, vomiting	[69, 82]
Oxcarbazepine	Block sodium channels	Simple partial seizures, complex partial seizures	Rash, hyponatremia	[70, 83]
Valproic Acid	Block sodium channels	Generalized and partial seizures	Weight gain, hair loss, hepatotoxicity, pancreatitis, gastritis	[68, 84]
Lamotrigine	Block sodium channels modulate calcium channels (N- and P/Q-type) and HCN channels	Refractory partial epilepsy, absence seizures, generalized seizures, and Lennox-gestalt syndrome	Steven’s Johnsson syndrome; dizziness, diplopia, sedation, skin rashes, blurred vision	[74, 85]
Levetiracetam	Bind to the SV2A protein	Focal and secondarily generalized tonic-clonic seizures and absence seizures	Depression, Somnolence, dizziness, asthenia, sedation	[80, 81]
Zonisamide	Block sodium channels and T-type calcium currents Block sodium	Partial seizures	Weight loss, somnolence, dizziness, nausea, headache	[73]
Topiramate	Block sodium channel, enhance GABA inhibition by GABA_A_ receptors, modulate calcium and potassium channels, the antagonistic effect on AMPA/kainate subtype of glutamate receptor	Partial-onset or primary generalized tonic-clonic seizures, Lennox-Gastautsyndrom	Cognitive impairment, weight loss, sedation, dizziness,	[83, 86]
Lacosamide	Block sodium channels and T-type calcium currents	Partial-onset seizures	Dizziness, abnormal vision, diplopia and ataxia	[71]
Lorazepam	Increase the frequency of opening of the chloride channel of the GABA_A_ receptor	Status epilepticus	Respiratory arrest, amnesia, dizziness, ataxia, confusion, sedation, depression, and tachycardia	[79, 87]

**Table 2:** JAKs and STATs that are activated by cytokines.

**Type I Cytokines**	**JAKs**	**STATs**
**Cytokines Whose Receptors Share γc**		
IL-2, IL-7, IL-9, IL-15, IL-21	JAK1, JAK3	STAT5, STAT3
IL-4	JAK2	STAT3, STAT5
**Cytokines Whose Receptors Share βc**		
IL-3, IL-5	JAK2	STAT3, STAT5
GM-CSF	JAK2	STAT3, STAT5
**Cytokines Whose Receptors Share gp130**		
IL-6, IL-11	JAK1, JAK2, TYK2	STAT1, STAT3
IL-12	JAK1, TYK2	STAT4
IL-23	JAK2, TYK2	STAT3, STAT4
IL-27	JAK1, JAK2, TYK2	STAT1, STAT2, STAT3, STAT4, STAT5
GH	JAK2	STAT3, STAT5a
EPO	JAK2	STAT5
TPO	JAK2	STAT1, STAT3, STAT5
Leptin	JAK2	STAT3, STAT5a
G-CSF	JAK2	STAT5
**Type II Cytokines**		
IFNα/β	JAK1, TYK2	STAT1, STAT2, STAT3, STAT4
IFNγ	JAK1, TYK2	STAT1, STAT2, STAT3, STAT4
IL-10	JAK1, TYK2	STAT1,STAT3,STAT5
IL-19	JAK1, JAK2, TYK2	STAT3
IL-20	JAK1, JAK2, TYK2	STAT3
IL-22	JAK1, JAK2, TYK2	STAT1, STAT3, STAT5
IL-24	JAK1	STAT3
IL-28	JAK1, TYK2	STAT1, STAT2, STAT3, STAT4, STAT5
IL-29	JAK1, TYK2	STAT1, STAT2, STAT3, STAT4, STAT5

## LIST OF ABBREVIATIONS

ADAM22: A Disintegrin and Metalloproteinase Domain-Containing Protein 22
ADAM23: A Disintegrin and Metalloproteinase Domain-Containing Protein 23
AEDs: Antiepileptic Drugs
BBB: Blood-brain Barrier
BDNF: Brain-derived Neurotrophic Factor
BRD2: Bromodomain Containing 2
CBZ: Carbamazepine
CCD: Coiled-coil Domain
CD: Crohn's Disease
CIS: Cytokine-induced SH2 Domain Protein
CML: Chronic Myeloid Leukemia
CNS: Central Nervous System
CSF: Cerebrospinal Fluid
CWE: Children With Epilepsy
DBD: DNA-binding Domain
dODN: Decoy Oligodeoxynucleotide
EEG: Electroencephalography
eEPSC: Evoked Excitatory Postsynaptic Current
EGF: Epidermal Growth Factor
FERM: Four-point-one protein, Ezrin, Radixin, and Moesin Domain
FS: Febrile Seizures
G-CSF: Granulocyte Colony-stimulating Factor
GABA: γ-aminobutyric Acid
GABA_A_R: GABA Type A Receptor
GAD: Glutamic Acid Decarboxylase
GBP: Gu/Rh-II Binding Protein
GH: Growth Hormone
Glu: Glutamate
GM-CSF: Granulocyte-macrophage Colony-stimulating Factor
GnRH: Gonadotropin-releasing Hormone
GVHD: Graft-*versus*-host Disease
HCN: Hyperpolarization-activated Cyclic Nucleotide-gated
IBD: Inflammatory Bowel Disease
IFN-I: Interferon-I
IFN-α: Interferon-α
IFN-β: Interferon-β
IFN-γ: Interferon-γ
IgG: Immunoglobulin G
IL-1: Interleukin-1
IL-1β: Interleukin-1 β
IL-2: Interleukin-2
IL-3: Interleukin-3
IL-6: Interleukin-6
ILAE: International League Against Epilepsy
ILCs: Innate Lymphoid Cells
IMD: Inherited Metabolic Disorders
IPSCs: Inhibitory Postsynaptic Currents
IVIg: Intravenous Immunoglobulins
JAK: Janus Kinase
JH: JAK Homology
KA: Kainic Acid
KL: Kinase-like
LD: Linker Domain
LGI1: Leucine-rich Glioma-inactivated 1
LncRNA: Long Non-coding RNA
LPS: Lipopolysaccharide
LTD: Long-term Inhibition
LTP: Long-term Potentiation
MRI: Magnetic Resonance Imaging
ND: N-terminal Domain
NELF: Nasal Embryonic Luteinizing Hormone-Releasing Factor
NK: Natural Killer
NMDA: N-methyl-D-aspartate
PDGF: Platelet-derived Growth Factor
PGE2: Prostaglandin E2
PHT: Phenytoin
PIAS: Protein Inhibitor of Activated STAT
PICs: Proinflammatory Cytokines
PSD: Postsynaptic Density
PTCL: Peripheral T-cell Lymphoma
PTK: Protein Tyrosine Kinases
PTPs: Protein Tyrosine Phosphatases
RA: Rheumatoid Arthritis
SH2: Src Homology 2
shRNA: Short Hairpin RNA
SLE: Systemic Lupus Erythematosus
SOCS: Suppressor of Cytokine Signaling
STAT: Signal Transducer and Activator of Transcription
T1DM: Type 1 Diabetes Mellitus
TAD: Transcriptional Activation Domain
TGF-β: Transforming Growth Factor-β
Th: T-helper
TLE: Temporal Lobe Epilepsy
TNBS: 2,4,6-Trinitrobenzene Sulfonic
TNF: Tumor Necrosis Factor
Treg: Regulatory T
UC: Ulcerative Colitis
UCA1: Urothelial Carcinoma-associated 1
VPA: Valproic Acid
ZNS: Zonisamide
